# Ecophysiology and Comparative Genomics of *Nitrosomonas mobilis* Ms1 Isolated from Autotrophic Nitrifying Granules of Wastewater Treatment Bioreactor

**DOI:** 10.3389/fmicb.2016.01869

**Published:** 2016-11-22

**Authors:** Soe Myat Thandar, Norisuke Ushiki, Hirotsugu Fujitani, Yuji Sekiguchi, Satoshi Tsuneda

**Affiliations:** ^1^Tsuneda Laboratory, Department of Life Science and Medical Bioscience, Waseda UniversityTokyo, Japan; ^2^Department of Biotechnology, Mandalay Technological University, Ministry of EducationMandalay, Myanmar; ^3^Advanced Biomeasurements Research Group, Biomedical Research Institute, National Institute of Advanced Industrial Science and TechnologyIbaraki, Japan

**Keywords:** nitrification, ammonia oxidation, wastewater, kinetics, *Nitrosomonas*, physiological characteristics, genomic properties

## Abstract

Ammonia-oxidizing bacteria (AOB), which oxidize ammonia to nitrite in the first step of nitrification, play an important role in biological wastewater treatment systems. *Nitrosomonas mobilis* is an important and dominant AOB in various wastewater treatment systems. However, the detailed physiological and genomic properties of *N. mobilis* have not been thoroughly investigated because of limited success isolating pure cultures. This study investigated the key physiological characteristics of *N. mobilis* Ms1, which was previously isolated into pure culture from the nitrifying granules of wastewater treatment bioreactor. The pure culture of *N. mobilis* Ms1 was cultivated in liquid mineral medium with 30 mg-N L^-1^ (2.14 mM) of ammonium at room temperature under dark conditions. The optimum growth of *N. mobilis* Ms1 occurred at 27°C and pH 8, with a maximum growth rate of 0.05–0.07 h^-1^, which corresponded to a generation time of 10–14 h. The half saturation constant for ammonium uptake rate and the maximum ammonium uptake rate of *N. mobilis* Ms1 were 30.70 ± 0.51 μM NH_4_^+^ and 0.01 ± 0.002 pmol NH_4_^+^ cells^-1^ h^-1^, respectively. *N. mobilis* Ms1 had higher ammonia oxidation activity than *N. europaea* in this study. The oxygen uptake activity kinetics of *N. mobilis* Ms1 were K_m(O_2_)_ = 21.74 ± 4.01 μM O_2_ and V _max(O_2_)_ = 0.06 ± 0.02 pmol O_2_ cells^-1^ h^-1^. Ms1 grew well at ammonium and NaCl concentrations of up to 100 and 500 mM, respectively. The nitrite tolerance of *N. mobilis* Ms1 was extremely high (up to 300 mM) compared to AOB previously isolated from activated sludge and wastewater treatment plants. The average nucleotide identity between the genomes of *N. mobilis* Ms1 and other *Nitrosomonas* species indicated that *N. mobilis* Ms1 was distantly related to other *Nitrosomonas* species. The organization of the genes encoding protein inventory involved in ammonia oxidation and nitrifier denitrification processes were different from other *Nitrosomonas* species. The current study provides a needed physiological and genomic characterization of *N. mobilis*-like bacteria and a better understanding of their ecophysiological properties, enabling comparison of these bacteria with other AOB in wastewater treatment systems and natural ecosystems.

## Introduction

Nitrification, the oxidation process of ammonia and nitrite followed by denitrification, is an important process in the nitrogen cycle of natural ecosystems and in biological wastewater treatment systems. This process is mainly performed by two groups of nitrifying microbes, ammonia-oxidizing bacteria and archaea (AOB and AOA), which convert ammonia (NH_3_) to nitrite (NO_2_^-^) in two steps, and nitrite-oxidizing bacteria (NOB) that oxidize the nitrite (NO_2_^-^) to nitrate (NO_3_^-^). Recently, some species belonging to lineage II of the genus *Nitrospira* in the NOB group have been reported as complete ammonia oxidizing bacteria (comammox) that perform the complete nitrification of ammonia to nitrate (Daims et al., 2015; van Kessel et al., 2015).

Because of the important role of nitrification in both the natural environment and wastewater treatment systems, researchers have long been trying to understand the ecophysiology of nitrifying microorganisms. Studies on nitrifying microbes in wastewater treatment systems and the natural environment using molecular methods have indicated that *Nitrosomonas* species are the main AOB populations in wastewater treatment bioreactors (Tsuneda et al., 2003; Chen and Wong, 2004; Mota et al., 2005; Wells et al., 2009), as well as in diverse natural environments including freshwater, coastal and brackish water regions (Stehr et al., 1995; Zhang et al., 2015). Among *Nitrosomonas* species, *Nitrosomonas europaea*/*Nitrosomonas mobilis* cluster 7 was dominant in wastewater treatment bioreactors loaded with high concentrations of ammonia and nitrite, whereas *Nitrosomonas oligotropha* cluster 6a was dominant in systems with a lower ammonia environment (Suwa et al., 1994, 1997; Bollmann and Laanbroek, 2001; Limpiyakorn et al., 2005, 2007; Tan et al., 2008). *Nitrosomonas mobilis*-like bacteria were codominant with *N. europaea*-*eutropha*- and *N. oligotropha*-like bacteria in the biofilm’s microbial populations in wastewater treatment bioreactors (Gieseke et al., 2003). In a study of nitrifying granules, *N. mobilis*-like bacteria were predominant from the nascent stage into maturity where *N. europaea*- and *N. oligotropha*-like bacteria were also present (Matsumoto et al., 2010). *N. mobilis* was also dominant in various wastewater treatment bioreactors (Juretschko et al., 1998; Chen and Wong, 2004; Mota et al., 2005). Because of this environmental distribution, *N. mobilis* has received a great deal of attention in the field of wastewater treatment.

The first isolation of *Nitrosococcus mobilis* from brackish water had been reported by Koops et al. (1976). It was later proposed that *Nitrosococcus mobilis* should be moved into the genus *Nitrosomonas* (Head et al., 1993). The original isolates (strains Nc2^T^, Nc3, Nc8) of *N. mobilis* were from brackish water, but a few environmental clones and pure strains (Nm 93, Nm104, Nm107) of *N. mobilis* have also been isolated from activated sludge samples (Koops and Harms, 1985; Juretschko et al., 1998; Purkhold et al., 2000). Studies of pure cultures and environmental clones of *N. mobilis* have been included in some phylogenetic analyses (Koops and Harms, 1985; Head et al., 1993; Pommerening-Roser et al., 1996; Juretschko et al., 1998; Purkhold et al., 2000; Cantera and Stein, 2007) and investigations of gene expression response to copper and zinc (Radniecki and Ely, 2008, 2011). According to Campbell et al. (2011), *Nitrosococcus mobilis* was published as *Nitrosomonas mobilis* with the type strain Nc2. However, to date, no representative strains of *N. mobilis* suitable for examination of their physiological and genomic characteristics have been reported and detailed information regarding the physiological and genomic characterization of *N. mobilis* is still lacking because of the limited success in isolating it into pure cultures.

Recently, the pure culture of *Nitrosomonas mobilis* Ms1 has been successfully isolated from the autotrophic nitrifying granules by selective isolation using a cell-sorting system (Fujitani et al., 2015). These nitrifying granules were produced in the aerobic upflow fluidized bed reactor to treat inorganic wastewater and exhibited an effective ammonia removal rate (Tsuneda et al., 2003). During the granule formation, *Nitrosomonas* species and *Nitrosomonas mobilis* act as an important and dominant AOB population (Tsuneda et al., 2003; Matsumoto et al., 2010). To accomplish an ecophysiological study of *N. mobilis* and related *Nitrosomonas* species, in this study we investigated key physiological characteristics such as temperature and pH ranges, growth rate, NaCl tolerance, ammonium and oxygen uptakes activity kinetics, ammonium substrate inhibition and nitrite tolerance of *N. mobilis* Ms1. In addition, genome sequencing and annotation of the genes were conducted. The genes encoding proteins involved in ammonia oxidation and nitrogen oxide metabolism were also identified. This study is the first physiological and genomic characterization of *Nitrosomonas mobilis* and provides important information regarding *N. mobilis*-like strains.

## Materials and Methods

### Cell Cultures, Media, and Cultivation

*Nitrosomonas mobilis* Ms1 isolated from nitrifying granules (Fujitani et al., 2015) was kept in liquid culture of mineral medium containing 30 mg-N L^-1^ of ammonium substrate (NH_4_Cl) with the following components per liter of distilled water: NaCl (0.116 g), MgSO_4_⋅7H_2_O (0.4 g), CaCl_2_⋅2H_2_O (0.073 g), KCl (0.038 g), KH_2_PO_4_ (0.034 g), FeCl_2_ (0.002 g), EDTA (0.0043 g), MnCl_2_⋅4H_2_O (0.1 mg), CoCl_2_⋅6H_2_O (0.024 mg), NiCl_2_⋅6H_2_O (0.024 mg), CuCl_2_⋅2H_2_O (0.017 mg), ZnCl_2_ (0.068 mg), Na_2_WO_4_⋅2H_2_O (0.033 mg), Na_2_MoO_4_ (0.024 mg), and H_3_BO_3_ (0.062 mg). The pH of the culture media was adjusted to 8.0 by NaHCO_3_ (0.84 g L^-1^), and phenol red (0.4 w/v %) was used as the pH indicator. The cultures were incubated under static and dark conditions at room temperature. For physiological and kinetics studies, mineral media with different ammonium concentrations was used and phenol red was omitted. To determine the effect of NaCl concentration, mineral media without NaCl was used. *Nitrosomonas europaea* Winogradsky 1892 (NBRC 14298) was purchased from the Biological Resource Center (NBRC), National Institute of Technology and Evaluation (NITE). The HEPES medium 829 as described in the manual NBRC No. 14298 was used as the growth medium in this study. For the activity experiment, media with separately prepared different concentrations of ammonium substrate [(NH_4_)_2_SO_4_] was used. The stock cultures of *N. mobilis* Ms1 and *N. europaea* were periodically checked for their culture conditions (pH, ammonium and produced nitrite concentrations), cell growth and morphologies, and transferred into fresh culture media when the nitrite concentrations in media reached approximately 200 mg-N L^-1^.

### Fluorescence Microscopy

The growth and morphology of the cell cultures during the experiments were observed by fluorescence microscopy. Briefly, 10 μL cell samples were applied to each well of 12-well glass slides and allowed to dry. The samples were then dried and stained with SYTOX Green nucleic acid stain (Life Technologies, Molecular Probes, Eugene, OR, USA) and observed under a fluorescence microscope (Zeiss Axioskop 2plus, lens Zeiss Plan-APOCHROMAT 100×/1.4 oil, Carl Zeiss, Oberkochen, Germany). To count the cell numbers, 1 mL of culture samples were sonicated at intensity of 4 for 1 min (Sonifier II model, Branson, Danbury, CT, USA) and heated at 95°C for 10 min, after which, 1 μL cell samples were applied to each well of the 12-well glass slides. The cell numbers were quantitatively analyzed from each well using a microscopic direct counting method. The mean cell numbers were determined from triplicate wells for each sample.

### Measurement of Ammonium and Nitrite Concentrations

The samples for measurement were collected by filtration through a 0.22 μm Micropore filter (Millex, Merck Millipore, Darmstadt, Germany), after which the ammonium and nitrite concentrations of collected samples were measured colorimetrically with a modified indophenol reaction (Kandeler and Gerber, 1988) at an absorbance wavelength of 630 nm and Griess Reagent (Griess-Romijn van Eck, 1966) at an absorbance wavelength of 550 nm, respectively, using a Powerscan HT microplate spectrophotometer (BioTek, Winooski, VT, USA).

### Effect of Temperature, pH and NaCl Concentration on the Growth of Ms1

Batch cultures experiments were conducted to investigate the effects of temperature, pH and NaCl concentrations on the growth of *N. mobilis* Ms1. The culture sample was concentrated by centrifugation at 4,800 × *g* and 23°C for 30 min. The sample pellet was then mixed with fresh mineral medium containing 30 mg-N L^-1^ (2.14 mM) initial ammonium substrate (NH_4_Cl) and subjected to weak sonication. To investigate the effect of NaCl concentration, fresh mineral media containing different NaCl concentrations (0, 50, 100, 250, 500, 750, and 1,000 mM) were used. To investigate the effects of pH, the culture media were adjusted to a pH of 4, 5, 6, 7, 8, 9, 11, or 13 by NaOH and HCl, then mixed with the sample pellet and subjected to weak sonication. Next, the cultures were incubated in rotatory shaker incubators at different temperatures (7–47°C, pH 8.0), pHs (pH 4–13, 27°C) and NaCl concentrations (NaCl 0–1,000 mM, 27°C, pH 8.0) for 24 h. Aliquots of samples (1–2 mL) were taken out at the start of the incubation period (0 h), 1 h (for the temperature experiments because the culture samples were assumed to have adapted to each temperature at this point) and the end of the incubation (24 h), then collected by filtration through a 0.22 μm Micropore filter (Millex). The growth of *N. mobilis* Ms1 was measured based on the nitrite concentrations of the collected samples. All experiments were conducted in triplicate.

### Maximum Specific Growth Rate (μ_max_)

The growth rate of *N. mobilis* Ms1 was studied by batch culture of 5 mL aliquots from pure cultures of *N. mobilis* Ms1 with 100 mg-N L^-1^ (7.14 mM) initial ammonium substrate in 100 mL fresh mineral media using 500 mL experimental flasks. The initial pH value of the batch cultures was 8.0 and the initial cell concentrations were approximately 1.64 × 10^4^–3.73 × 10^5^ cells mL^-1^. The cultures were incubated at 27°C on a rotatory shaker incubator for 1 week until all ammonium was consumed. During the incubation period, the pH of the culture was kept constant at around 8.0 by adding sterile NaHCO_3_. The aliquots of samples (1–2 mL) were taken out daily at the same time from the start until the end of the incubation period and collected by filtering through a 0.22 μm Micropore filter (Millex) to determine the ammonium and nitrite concentrations. The cell numbers were also determined by direct microscopic counting with Sytox green staining. The specific growth rate (μ) and generation time (g) were then calculated using the following equations:

(1) $$\mu =\frac{\mathrm{Ln}(X_{2}{-X}_{1})}{\left(t_{2}{-t}_{1}\right)}$$

(2) $$g=\frac{\mathrm{Ln}(2)}{\mu }$$

where X is the number of cells and t is the time (Belser and Schmidt, 1980). All experiments were conducted in duplicate.

### Activity Kinetics

The activity kinetics of *N. mobilis* Ms1 were investigated by measuring the ammonium and oxygen uptake activities. To determine the ammonium uptakes, short-term incubation in a batch culture was conducted. The culture sample of *N. mobilis* Ms1 was concentrated by centrifugation at 4,800 × *g* and 23°C for 30 min. The sample pellet was mixed with 100 mL of fresh mineral medium containing 7.0 mg-N L^-1^ (500 μM) of ammonium and subjected to weak sonication. The cell concentrations of the experimental cultures were approximately 1.31 × 10^7^–2.82 × 10^7^ cells mL^-1^. The ammonium uptake activity kinetics of *N. europaea* were also studied for comparison with those of *N. mobilis* Ms1. The culture sample of *N. europaea* was concentrated as in *N. mobilis* Ms1 and mixed with 100 mL fresh mineral medium containing 5.0 mg-N L^-1^ (357.14 μM). The pH of the media was then adjusted to around 8.0 by NaHCO_3_. Next, samples were incubated at 27°C in a rotatory shaker incubator for about 3–6 h until all ammonium was consumed, during which time aliquots of samples (1–2 mL) were taken out every 10 min, filtered through a 0.22 μm Micropore filter (Millex), and measured for ammonium concentration. The cell numbers in the aliquots of the samples collected at 0 h were also counted. The ammonium concentrations obtained over time and the ammonium uptake rates were fitted to the Michaelis–Menten equation, which is as follows:

(3) $$V=\frac{\left(V_{\max\;({\mathrm{NH}}_{4}^{+})\;\times }[{\mathrm{NH}}_{4}^{+}]\right)}{\left(K_{m\;({\mathrm{NH}}_{4}^{+})}+[{\mathrm{NH}}_{4}^{+}]\right)}$$

where *V* is the ammonium uptake rate, V _max ($NH_{4}^{+}$)_ is the maximum ammonium uptake rate, K_m ($NH_{4}^{+}$)_ is the half saturation constant for ammonium uptake, and [NH_4_^+^] is the ammonium concentration. V _max ($NH_{4}^{+}$)_ was calculated as the ammonium uptake rate per cell concentration. All experiments were conducted in duplicate. The specific affinity for ammonium was calculated using the ratio of kinetics values from ammonium uptake activity (i.e., V _max ($NH_{4}^{+}$)_ × [K_m ($NH_{4}^{+}$)_]^-1^) (Laanbroek and Gerards, 1993).

An O_2_ microsensor (OX-MR13831, Unisense, Aarhus, Denmark) was used to measure the oxygen uptake of *N. mobilis* Ms1. The chamber and sensor were assembled in the water bath, and the temperature of the water bath was set to 30°C, at which time the sensor was calibrated. Next, 2 mL aliquots from *N. mobilis* Ms1 culture containing with 2.35 × 10^6^–5.20 × 10^6^ cells mL^-1^ were added into the chamber with a glass-coated magnet stirrer and the O_2_ sensor was immersed in the sample. When the O_2_ concentration was stable, measurement of the oxygen levels in the cell culture started. The data (oxygen concentrations over time) obtained by the O_2_ sensor were analyzed and smoothed using the SigmaPlot software (SigmaPlot 13.0, Systat software GmbH, Erkrath, Germany). Subsamples from the cell culture were used to count the cell numbers by direct microscopy after Sytox green staining. K_m (O_2_)_ and V _max (O_2_)_ were calculated from the measured oxygen uptake rate and the counted cell numbers using the Michaelis–Menten equation:

(4) $$V=\frac{\left(V_{\max\;(O_{2})}\times [O_{2}]\right)}{\left(K_{m\;(O_{2})}+[O_{2}]\right)}$$

where *V* is the oxygen uptake rate, V _max (O_2_)_ is the maximum oxygen uptake rate, K_m (O_2_)_ is the half saturation constant for oxygen uptake rate, and [O_2_] is the oxygen concentration. All experiments were conducted in triplicate.

### Ammonium and Nitrite Tolerance

To investigate the ammonium and nitrite tolerance of *N. mobilis* Ms1, batch experiments were conducted using different ammonium and nitrite concentrations. *N. mobilis* Ms1 was cultivated in 100 mL of fresh mineral media using 500 mL flasks containing different initial ammonium concentrations (10, 50, 100, 200, 300, 400, and 500 mM) and nitrite concentrations (0, 5, 10, 50, 100, 200, 300, 400, and 500 mM) in which an initial ammonium concentration of 5 mM was contained. The pH of all media was adjusted to 8.0 by NaHCO_3_. Cultures were incubated at 27°C in a rotatory incubator until all ammonium was consumed. During the experiment, aliquots of samples (1–2 mL) were collected at the start of the incubation period and every other day from 10 to 100 mM ammonium and 0–100 mM nitrite cultures, and every 4th day from 200 to 500 mM ammonium and nitrite cultures by filtering with a 0.22 μm Micropore filter (Millex). The initial ammonium concentrations and produced nitrite concentrations of the collected samples were measured colorimetrically by indophenol and Griess Reagent tests, respectively. During the incubation period, the cell growth was observed by Sytox green fluorescence microscopic examination of the collected samples and the pHs of the media were kept constant at around 8.0 by adding sterile NaHCO_3_. All experiments were conducted in duplicate.

### Genome Sequencing, Assembly and Gene Annotation

A draft genome sequence of strain Ms1 was performed as follows. Briefly, genomic DNA was extracted from a pure culture of strain Ms1 by using NucleoSpin^®^ Tissue, a DNA extraction kit (Takara Bio, Otsu, Japan) according to the manufacturer’s instructions, and a Nextera XT paired-end (300–1,000 bp) was prepared from the extracted DNA. The library was then sequenced on an Illumina MiSeq instrument with V2 chemistry (2 × 250 bp reads) at an expected coverage of 120× per genome. Raw sequence reads were merged with SeqPrep with concurrent removal of sequencing adaptors, followed by quality filtering and trimming of the unmerged reads with Nesoni version 0.112. Both merged and processed unmerged reads were combined for assembly using SPAdes version 2.5.0 (Bankevich et al., 2012), followed by manual improvement of the assembly, as described previously (Sekiguchi et al., 2015). The final draft genome assembly of the Ms1 genome consists of 113 contigs. The largest and N50 contig sizes are 251,952 and 65,925 bp, respectively. The total assembly size is 3,083,004 bp. Annotation was performed with MicroScope microbial genome annotation and analysis platform (Vallenet et al., 2013). Coding sequences (CDS) were automatically predicted, annotated and categorized by clusters of orthologous groups (COGs) using MicroScope platform. The contigs of genome sequences of *N. mobilis* Ms1 have been deposited into European Nucleotide Archive (ENA) database under the accession number FMWO01000001-FMWO01000112 (BioProject number PRJEB15545).

### Average Nucleotide Identity Analysis

The average nucleotide identity (ANI) analysis was conducted to determine whether *N. mobilis* Ms1 represents distant AOB species (Konstantinidis and Tiedje, 2005). The genome sequence of *Nitrosomonas mobilis* Ms1 was compared with the genome sequences of *N. europaea* ATCC 19718, *N. eutropha* C 71, *Nitrosomonas* sp. AL212, and *Nitrosomonas* sp. Is79A3. The ANI values between Ms1 and other genomes were calculated using EzGenome provided online by Jongsik Chun, Chunlab, Inc^1^.

## Results and Discussion

### Physiological Characteristics of *N. mobilis* Ms1

#### Effect of Temperature, pH and NaCl Concentration

**FIGURE 1:**
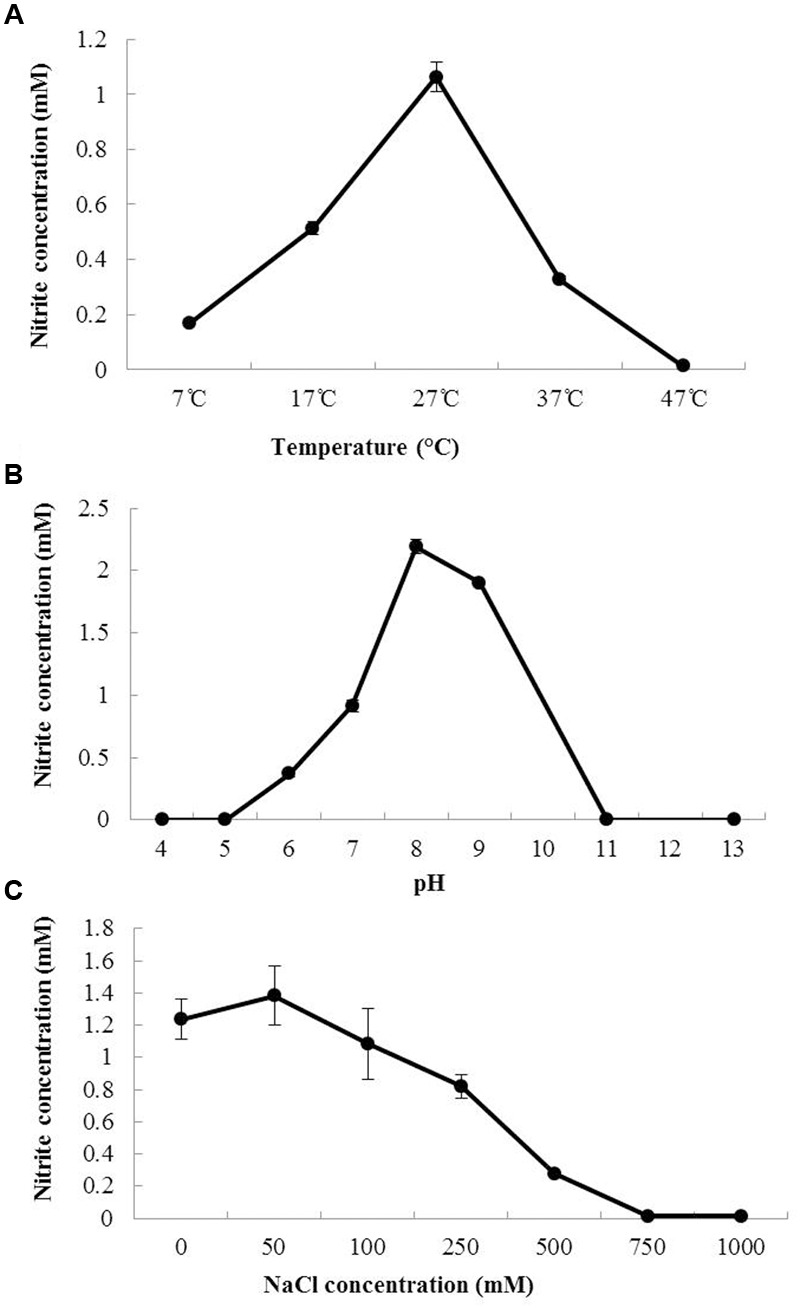
**Effect of temperature, pH and NaCl concentration on the growth of *N. mobilis* Ms1.** The nitrite concentrations of *N. mobilis* Ms1 after 24 h of incubation at different temperatures **(A)**, pHs **(B)**, and NaCl concentrations **(C)**. Bars are the standard errors (*n* = 3).

Batch experiments were conducted to investigate the effect of temperature (**Figure 1A**), pH (**Figure 1B**) and NaCl concentration (**Figure 1C**) on the growth of *N. mobilis* Ms1. The growth and ammonia oxidation activity were confirmed by the nitrite concentrations of the culture samples. *N. mobilis* Ms1 has a temperature range of 17–37°C, with an optimum temperature of 27°C, which is similar to the optimal ranges of other pure culture AOB strains such as *Nitrosomonas europaea* ATCC 25978^T^ (25–29°C), *Nitrosomonas eutropha* C-91^T^ (30°C), *Nitrosococcus mobilis* Nc2^T^ (25–30°C) in the *Nitrosomonas europaea*/*Nitrosomonas mobilis* lineage, as well as *Nitrosomonas* sp. strain AL212 (25°C) in the *Nitrosomonas oligotropha* lineage, *Nitrosomonas* sp. JPCCT2 (28°C) in the *Nitrosomonas communis* lineage and *Nitrosomonas stercoris* KYUHI-S^T^ (25°C) in an unclassified cluster (**Table 1**) (Koops et al., 1976, 1991; Suwa et al., 1997; Tokuyama et al., 1997; Itoh et al., 2013; Nakagawa and Takahashi, 2015). *N. mobilis* Ms1 oxidized well at pH 8.0, while other *Nitrosomonas* species showed their optimal oxidation at pH values ranging from 7.2 to 8.2 (**Table 1**). Similarly to *N. europaea* and *N. eutropha, N. mobilis* Ms1 required no salt and showed NaCl tolerance up to 500 mM (**Table 1**). Growth temperature and pH ranges, and salt requirement of each strain reflect the diversity and habitat of *Nitrosomonas* species in both man-made and natural ecosystems (Jones et al., 1988; Koops et al., 1991, 2006; Koops and Pommerening-Roser, 2001; Cortes-Lorenzo et al., 2015).

**Table 1:** Physiological characteristics of pure cultures of *N. mobilis* Ms1 and some AOB species.

Strains	*N. europaea/N. mobilis* lineage, cluster 7				*N. communis* lineage, cluster 8	*N. oligotropha* lineage, cluster 6a	Unclassified cluster
	*N. mobilis* Ms1	*N. europaea* ATCC19718^T^	*N. eutropha* C-91^T^	*N. mobilis* Nc2^T^	*Nitrosomonas* sp. strain JPCCT2	*Nitrosomonas* sp. strain AL212	*N. stercoris* KYUHI-S^T^
Temperature range (°C)	17–37	10–40^a^	NA	10–30	28–48	NA	20–37
Optimum temperature (°C)	27	25–29^a^	30	25–30	28	25	25
pH range (-)	7–9	NA	NA	6.8–8.3	4.7–8.3	NA	7–9
Optimum pH (-)	8.0	7.8–8.2^a^	7.5–8.0	7.5–7.8	7.5–8.0	7.2	8.0
Growth rate, μ_max_ (h^-1^)	0.06 ± 0.01	0.05–0.07	NA	0.053–0.058	NA	0.014–0.025^d^	0.029
Generation time (h)	10–14	10–14	NA	12–13	NA	28–49^d^	24
NaCl requirement	-	-	-	+	-	-	-
Maximum NaCl tolerance (mM)	500	400	400	500	300	NA	400
Half-saturation constant, K_m ($NH_{4}^{+}$)_ (μM NH_4_^+^)	30.70 ± 0.51	55.43 ± 4.92^b^	NA	NA	NA	NA	NA
Maximum ammonium uptake rate per cell, V _max ($NH_{4}^{+}$)_ (pmol NH_4_^+^ cells^-1^ h^-1^)	0.01 ± 0.002	0.0009 ± 0.00003^b^	NA	NA	NA	NA	NA
Half-saturation constant, K_m (O_2_)_ (μM O_2_)	21.74 ± 4.01	NA	NA	NA	NA	NA	NA
Maximum oxygen uptake rate per cell, V _max (O_2_)_ (pmol O_2_ cells^-1^ h^-1^)	0.06 ± 0.02	NA	NA	NA	NA	NA	NA
Maximum ammonium tolerance (mM)	100	400	600	300	NA	10.7	1000
Ammonium concentration in standard growth medium (mM)	2.14	10	10	9.34	11.25–15.0	3.57	38
Nitrite tolerance (mM)	300	20^c^	10	14.28	NA	2.1–5.4^e^	200
Origin	Nitrifying granules	Wastewater	Municipal sewage	Brackish water	Activated sludge	Activated sludge	Compost
Reference	This study	Belser and Schmidt, 1980; Koops et al., 1991, 2006; Tokuyama et al., 1997; Stein and Arp, 1998; Cua and Stein, 2011	Koops et al., 1991, 2006; Cua and Stein, 2011	Koops et al., 1976, 1990	Itoh et al., 2013	Suwa et al., 1994, 1997; Limpiyakorn et al., 2007	Nakagawa and Takahashi, 2015

*+, positive; -, negative; ^a^N. europaea ATCC25978^T^; ^b^this study; ^c^AMO enzyme inhibition (50%); ^d^the pure cultures of *N. oligotropha*-like bacteria; ^e^inhibitory range for *N. oligotropha*-like bacteria; ^T^type strain; NA, not available.*

#### Growth Rate and Activity Kinetics

**FIGURE 2:**
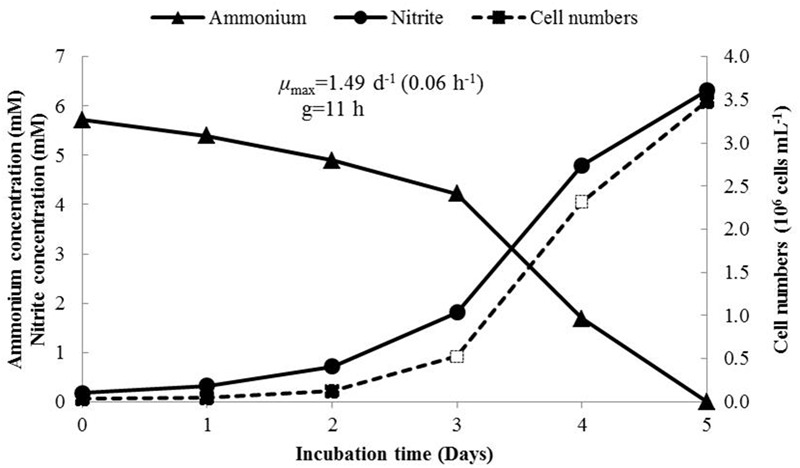
**Growth of *N. mobilis* Ms1 determined by measurement of time courses of ammonium concentrations (triangle), nitrite concentrations (circle) and cell numbers (rectangle) during incubation for 5 days.** The maximum growth rate calculated from the increased number of cells was observed on days 3 and 4 (open rectangle).

The growth rate of *N. mobilis* Ms1 was studied in batch cultures to calculate the maximum specific growth rate (μ_max_) and generation time (*g*). The initial cell concentrations were 4.07 × 10^4^ ± 9.87 × 10^3^ cells mL^-1^, and the highest number of cells reached 3.53 × 10^6^ ± 4.12 × 10^5^ cells mL^-1^ on day 5 of incubation. At this time, almost all ammonium in the cultures was consumed, the produced nitrite concentrations increased and ammonium concentrations decreased (**Figure 2**). Under the optimum conditions, the maximum specific growth rate (μ_max_) of *N. mobilis* Ms1 cells reached 0.06 ± 0.01 h^-1^ and the generation time (*g*) was 11.87 ± 2.33 h, which is in the range of previous reports for *Nitrosomonas* species within the *N. europaea/N. mobilis* lineage, as *Nitrosomonas europaea* and *Nitrosococcus mobilis* had generation times of 10–14 and 12–13 h, respectively (**Table 1**) (Koops et al., 1976; Belser and Schmidt, 1980). Generally, members in the *N. europaea/N. mobilis* lineage have a shorter generation time than other ammonia oxidizers. For example, members belonging to the *N. oligotropha* lineage and *Nitrosospira* lineage had generation times of 28–49 and 18–115 h, respectively, while the newly isolated highly ammonia-tolerant AOB strain, *N. stercoris* KYUHI-S^T^, belonging to an unclassified cluster, had a generation time of 24 h (Suwa et al., 1994; Bollmann and Laanbroek, 2001; Nakagawa and Takahashi, 2015).

**FIGURE 3:**
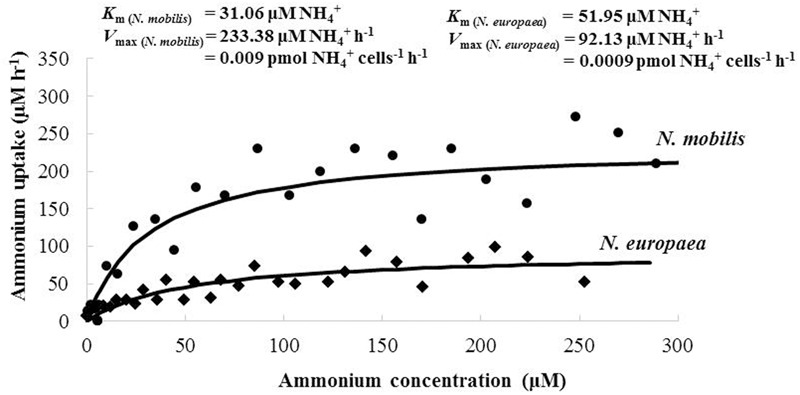
**Representative data describing ammonium uptake activities of *N. mobilis* Ms1 and *N. europaea*.** The best-fit curve was described according to the Michaelis–Menten equation to obtain K_m ($NH_{4}^{+}$)_ and V _max ($NH_{4}^{+}$)_ values.

The activity kinetics of *N. mobilis* Ms1 were investigated using the ammonium and oxygen uptake rates. To obtain a comparable data set for ammonia oxidation activity of *N. mobilis* Ms1, the same experiment was also conducted for *N. europaea*. The K_m ($NH_{4}^{+}$)_ and V _max ($NH_{4}^{+}$)_ values determined from the ammonium uptake rates of *N. mobilis* Ms1 and *N. europaea* were 30.70 ± 0.51 μM NH_4_^+^ and 0.01 ± 0.002 pmol NH_4_^+^ cells^-1^ h^-1^, and 55.43 ± 4.92 μM NH_4_^+^ and 0.0009 ± 0.00003 pmol NH_4_^+^ cells^-1^ h^-1^ (*n* = 2), respectively (**Figure 3**). According to the calculated specific affinity from the kinetics values of ammonium uptake activities, *N. mobilis* Ms1 has higher substrate affinity (0.33 ± 0.06 nL NH_4_^+^ cell^-1^ h^-1^) compared with *N. europaea* (0.016 ± 0.0008 nL NH_4_^+^ cell^-1^ h^-1^). This result is consistent with a previous study on microbial community structure in nitrifying granules, where the population of *N. mobilis* was larger compared with *N. europaea* when the ammonia concentration in the reactor reached almost zero (Matsumoto et al., 2010).

Based on our knowledge of *N. mobilis* Ms1, this strain has a high ammonia oxidation activity, i.e., Ms1 consumes ammonium quickly during both the cultivation period of stock cultures and the batch culture incubation for growth and activity measurements. These cultural and physiological findings were proved by the same experiment for ammonia oxidation activity of *N. mobilis* Ms1 and *N. europaea.* The V _max ($NH_{4}^{+}$)_ values of *N. mobilis* Ms1 (0.01 ± 0.002 pmol NH_4_^+^ cells^-1^ h^-1^) were higher than those of *N. europaea* (0.0009 ± 0.00003 pmol NH_4_^+^ cells^-1^ h^-1^). These finding are also supported by evidence of cultivation experiments for both strains, in which the ammonium concentration of 5.0 mg-N L^-1^ (357.14 μM) was completely consumed by *N. mobilis* Ms1 within an incubation time of 3 h, but consumed by *N. europaea* in 8 h, while their cell numbers were nearly same at 2.07 × 10^10^–9.55 × 10^10^ cells L^-1^. However, the activity kinetics of the ammonia oxidizers varied, even among the same species, for example, the activity of *N. europaea* ranged from 0.001 to 0.023 pmol cells^-1^ h^-1^ (Belser and Schmidt, 1980; Prosser, 1989).

**FIGURE 4:**
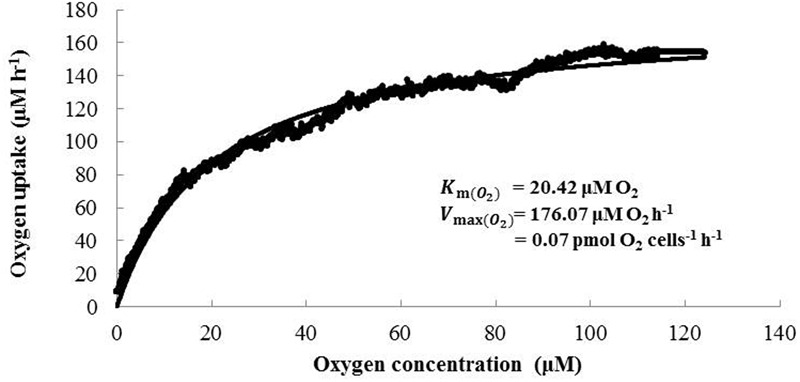
**Representative data describing oxygen uptake of *N. mobilis* Ms1.** The best-fit curve was described according to the Michaelis–Menten equation to obtain K_m_ (O_2_) and V_max (O_2_)_ values.

The kinetics values, K_m (O_2_)_ and V _max (O_2_)_, for the oxygen uptake rate of *N. mobilis* Ms1 were also determined and the results showed values of 21.74 ± 4.01 μM O_2_ and 0.06 ± 0.02 pmol O_2_ cells^-1^ h^-1^ (*n* = 3), respectively (**Figure 4**). This K_m (O_2_)_ value for *N. mobilis* Ms1 is comparable to those reported in a previous study for oxygen consumption kinetics of *N. europaea* ATCC 19178^T^ at a high growth rate and high oxygen concentration [K_m (O_2_)_ = 3.0–14.9 μM O_2_] (Laanbroek and Gerards, 1993). The respiratory cytochrome c oxidases of the nitrifiers are responsible for maintaining their electron chain and adapting to the environment at different oxygen levels (Klotz and Stein, 2011). *N. europaea* and *N. mobilis* Ms1 possess the cytochrome aa3 type oxidase, which functions as the low affinity O_2_ reductase whereas *N. eutropha* possesses cytochrome cbb3 type oxidase, which functions as the high affinity O_2_ reductase, in addition to cytochrome aa3 and bo3 type oxidases (Stein et al., 2007; Klotz and Stein, 2011). It is likely that *N. mobilis* Ms1 might not adapt to an environment at different oxygen levels.

#### Ammonium and Nitrite Tolerance

**FIGURE 5:**
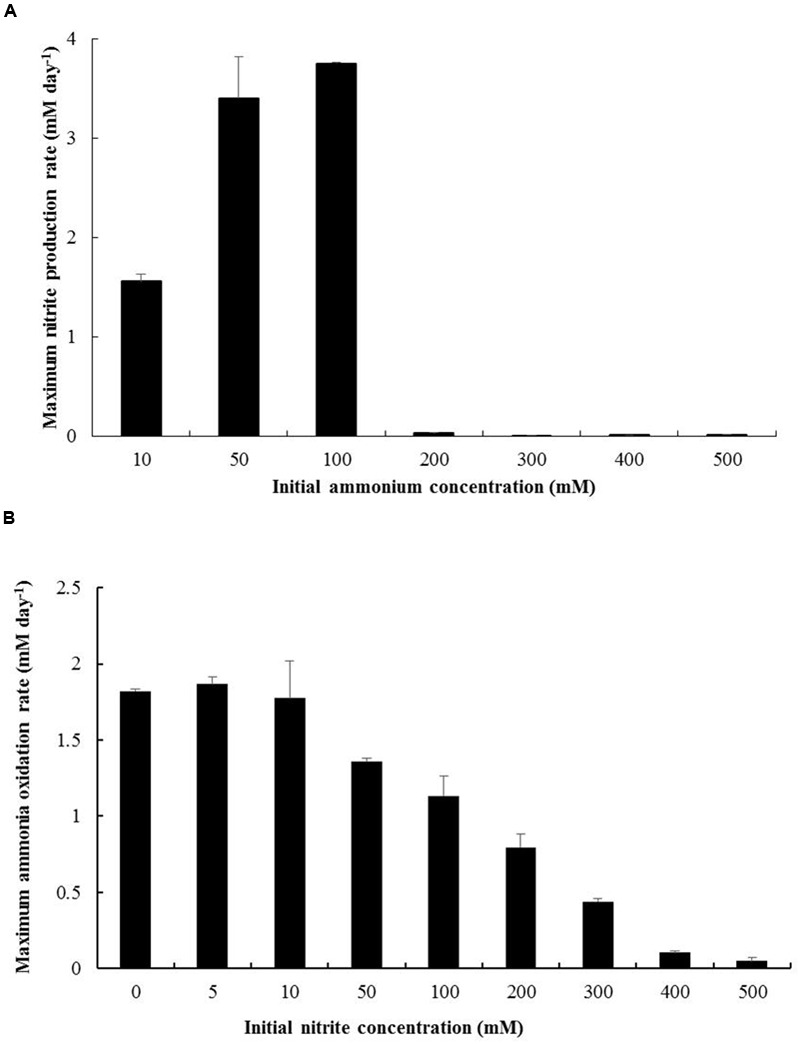
**Ammonium and nitrite tolerance of *N. mobilis* Ms1. (A)** Maximum produced nitrite concentration rate per day of *N. mobilis* Ms1 incubation with different initial ammonium concentrations (10, 50, 100, 200, 300, 400, and 500 mM). **(B)** Maximum ammonia oxidation rate per day of *N. mobilis* Ms1 incubation with different initial nitrite concentrations (0, 5, 10, 50, 100, 200, 300, 400, and 500 mM) under 5 mM initial ammonium concentration. Bars are the standard errors (*n* = 2).

*N. mobilis* Ms1 exhibits ammonia oxidizing ability at the concentrations of ammonium up to 100 mM (1,400 mg-N L^-1^) (**Figure 5A**). In tests of nitrite tolerance, although inhibition of ammonia oxidation was observed in cultures with concentrations of nitrite of 50 mM and higher (**Figure 5B**), the initially provided ammonium (5 mM) was finally consumed in cultures with nitrite concentrations of up to 300 mM. Taken together, *N. mobilis* Ms1 had high ammonium and nitrite tolerance, although the strain was enriched from the nitrifying granules of a low ammonium influent bioreactor and could grow in culture media with only 30 mg-N L^-1^ (2.14 mM) of ammonium substrate. These characteristics are consistent with those reported in a previous study by Juretschko et al. (1998), where *N. mobilis*-like bacteria were dominant in sludge with high ammonium concentrations of up to 5,000 mg-N L^-1^ (357.14 mM) in a wastewater treatment plant. Among members of the genus *Nitrosomonas, Nitrosomonas nitrosa* isolated from a eutrophic environment exhibited the same ammonium tolerance as *N. mobilis* Ms1, of up to 100 mM (Koops et al., 1991). In contrast, *Nitrosomonas* sp. strain AL212 belonging to the *N. oligotropha* lineage isolated from activated sludge exhibited the maximum ammonium tolerance of 10.7 mM (Suwa et al., 1997), which is about 10 times lower than that of *N. mobilis* Ms1 (100 mM). The results of the pure culture study also support the previous findings for AOB communities in which the *N. oligotropha* cluster was predominant over the *N. europaea-N. mobilis* cluster in activated sludge with low ammonium concentrations (Limpiyakorn et al., 2005, 2007). Within *Nitrosomonas* cluster 7, *Nitrosococcus mobilis* Nc2, which is phylogenetically similar to *N. mobilis* Ms1, was tolerant to ammonium concentration at up to 300 mM (Koops et al., 1990), while *N. europaea* and *N. eutropha*-like species were tolerant of up to 400 and 600 mM of ammonium, respectively (Koops et al., 1991). *Nitrosomonas stercoris* KYUHI-S^T^ isolated from compost (Nakagawa and Takahashi, 2015) exhibited the highest ammonium tolerance (1,000 mM) among *Nitrosomonas* strains (**Table 1**). Although the nitrite tolerance of ammonia oxidizers was not surprising because of the accumulation of nitrite during cultivation and enrichment, the 300 mM of nitrite tolerance of *N. mobilis* Ms1 was extremely high among strains isolated from the activated sludge and wastewater treatment plants. For example, the ammonia monooxygenase (AMO) activity of *N. europaea* was inhibited by 20 mM of nitrite (Stein and Arp, 1998; Cua and Stein, 2011). However, the high nitrite tolerance of *N. mobilis* Ms1 is consistent with the results of previous studies that showed enriched cultures of ammonia oxidizers had nitrite tolerance of up to 500 mM, most clones fell into the *Nitrosomonas europaea/Nitrosomonas mobilis* cluster (Tan et al., 2008), and nitrite effects on ammonia oxidizing activity varied between *Nitrosomonas europaea* and closely related *Nitrosomonas eutropha* (Cua and Stein, 2011).

### Genome Overview of *N. mobilis* Ms1

The genome of *N. mobilis* Ms1 has a sequence length of 3.09 Mbp and a G+C content of 48.53%. The genome included 3075 protein-coding DNA sequences (CDSs), 41 tRNA genes, and a single copy of the 16S-23S-5S rRNA operon. Based on phylogenetic analyses of *N. mobilis* Ms1 with other AOB (Fujitani et al., 2015), Ms1 showed the greatest similarity to *Nitrosomonas* sp. strains Nm107 and Nm104 (99.7–100%) at the 16S rRNA and *amoA* genes levels and 99.6% similarity to *Nitrosococcus mobilis* Nc2 at the 16S rRNA gene level, therefore Ms1 was attributed to the *N. mobilis* lineage. Similarities between *N. mobilis* Ms1 and other currently available genomes of *Nitrosomonas* species at 16S rRNA gene level and *amoA* amino acid level were highest to *Nitrosomonas europaea* ATCC 19718, 95.8 and 90.9%, respectively, and the similarity at the genomes level (ANI values) was highest to *N. eutropha* C-91, 71.1% (**Table 2**), confirming *N. mobilis* Ms1 was significantly below the species cut-off point for delineation (Konstantinidis et al., 2006). The smaller similarities of whole genomes and 16S rRNA and *amoA* sequences between *N. mobilis* Ms1 and other *Nitrosomonas* species support the physiological uniqueness of *N. mobilis* Ms1 among other *Nitrosomonas* species. The general genome features and the inventory of genes involved in the ammonia oxidation and nitrogen oxide metabolism of *N. mobilis* Ms1 and some currently available genomes of AOB are categorized in **Table 2**.

**Table 2:** Genome characteristics of *N. mobilis* Ms1 and some AOB species.

Characteristics	*N. mobilis* Ms1	*N. europaea* ATCC 19718	*N. eutropha* C71/C91	*Nitrosomonas* sp. Is79	*Nitrosomonas* sp. AL212
Genome size (bp)	3, 093,964	2,812,094	2,661,057 (2,781,824)^a^	3,783,444	3,180,526 (3,337,023)^a^
DNA G+C content (%)	48.5	50.7	48.5	45.4	44.7
CDSs	3075	2462	2551	3372	2983
rRNA (5S, 16S, 23S)	1, 1, 1	1, 1, 1	1, 1, 1	1, 1, 1	1, 1, 1
tRNA	41	41	41	38	38
16S rRNA similarity (DNA) (%)^†^	^†^	95.8	94.8	93.4	92.7
*amoA* similarity (DNA) (%)^†^	^†^	79.9	80.2	74.3	74.1
*amoA* similarity (amino acid) (%)^†^	^†^	90.9	89.8	80.3	80.3
ANI (%)^∗^	^∗^	70.7	71.1	67.8	67.5
Ammonia monooxygenase (*amoCAB*)	2 copies NSMM_350045-350048, NSMM_880001-880003	2 copies NE0945-0943, NE2064-2062	2 copies Neut_2078-2076, Neut_2319-2317	3 copies Nit79A3_0471-0473, Nit79A3_2886-2884, Nit79A3_1079-1081	3 copies NAL212_0797-0799, NAL212_1386- 1388, NAL212_2606- 2604
*amoC*	ND	1 copy NE1411	1 copy Neut_1520	2 copies Nit79A3_1233, Nit79A3_1595	2 copies NAL212_2303, NAL212_2818
ORF4 (*amoE*)	2 copies NSMM_880004, NSMM_350049	2 copies NE0942, NE2061	2 copies Neut_2075, Neut_2316	2 copies Nit79A3_0474, Nit79A3_2883	3 copies NAL212_0800, NAL212_2603, NAL212_2819
ORF5 (*amoD*)	1 copy NSMM_880005	2 copies NE0941, NE2060	2 copies Neut_2074, Neut_2315	2 copies Nit79A3_0475, Nit79A3_2882	2 copies NAL212_0801, NAL212_2602
Hydroxylamine oxidoreductase (*haoAB*)	2 copies NSMM_350001-350002, NSMM_410105-410106 2 *haoA* fragments NSMM_360037, NSMM_640011	3 copies NE0962-0961, NE2044-2043, NE2339-2338	3 copies Neut_1672-1671, Neut_1793-1792, Neut_2335-2334	3 copies Nit79A3_0807-0808, Nit79A3_0822-0823, Nit79A3_2942-2941	3 copies NAL212_1807-1806, NAL212_2138-2137, NAL212_2750-2749
Cytochrome c-554 (*cycA*)	2 copies NSMM_350003, NSMM_410107 2 *cycA* fragments NSMM_560024, NSMM_860012	3 copies NE0960, NE2042, NE2337	3 copies Neut_1670, Neut_1791, Neut_2333	3 copies Nit79A3_0809, Nit79A3_0824, Nit79A3_2940	3 copies NAL212_1805, NAL212_2136, NAL212_2748
Cytochrome c_M_-552 (*cycB/cycX*)	2 copies NSMM_560023, NSMM_860011	2 copies NE0959, NE2336	2 copies Neut_1790, Neut_2332	3 copies Nit79A3_0810, Nit79A3_0825, Nit79A3_2939	3 copies NAL212_1804, NAL212_2747, NAL212_2135
Nitrosocyanin	1 copy NSMM _680003	1 copy NE0143	1 copy Neut_2173	–	1 copy NAL212_0897
Copper-containing nitrite reductase, *nirK*	1 copy NSMM_600004	1 copy NE0924	1 copy Neut_1403	1 copy Nit79A3_2335	1 copy NAL212_2392
Nitric oxide reductase cNOR (*norCBQD*)	1 copy NSMM_630006- 630008, NSMM_400223	1 copy NE2003-2006	1 copy Neut_0518-0521	-	1 copy NAL212_0538-0541
Haem-copper nitric oxide reductase sNOR (*norSY-SenC*)	1 copy NSMM_310017-310019	1 copy NE0682-0684	1 copy Neut_1874-1876	-	-
Cytochrome c ′ beta (*cytS*)	1 copy NSMM_340048	1 copy NE0824	1 copy Neut_1345	1 copy Nit79A3_0363	1 copy NAL212_3151
Cytochrome P460 (*cytL*)	2 copies NSMM_320010 NSMM_810003	1 copy NE0011	1 copy Neut_0132	1 copy Nit79A3_1628	2 copies NAL212_0896, NAL212_0043
Origin	Nitrifying granules	Wastewater	Municipal sewage	Fresh water	Activated sludge
Bioproject accession number	PRJEB15545	PRJNA52	PRJNA13913	PRJNA52837	PRJNA32989
Reference	This study	Chain et al., 2003	Stein et al., 2007	Bollmann et al., 2013	Suwa et al., 2011

*^∗^Average nucleotide identity (ANI) of Ms1 with other AOB pure strains; ^†^percent (%) similarity of Ms1 with other AOB pure strains; -, absence of the gene in the genome; ^a^size including plasmids; ND, not detected in the genome.*

**FIGURE 6:**
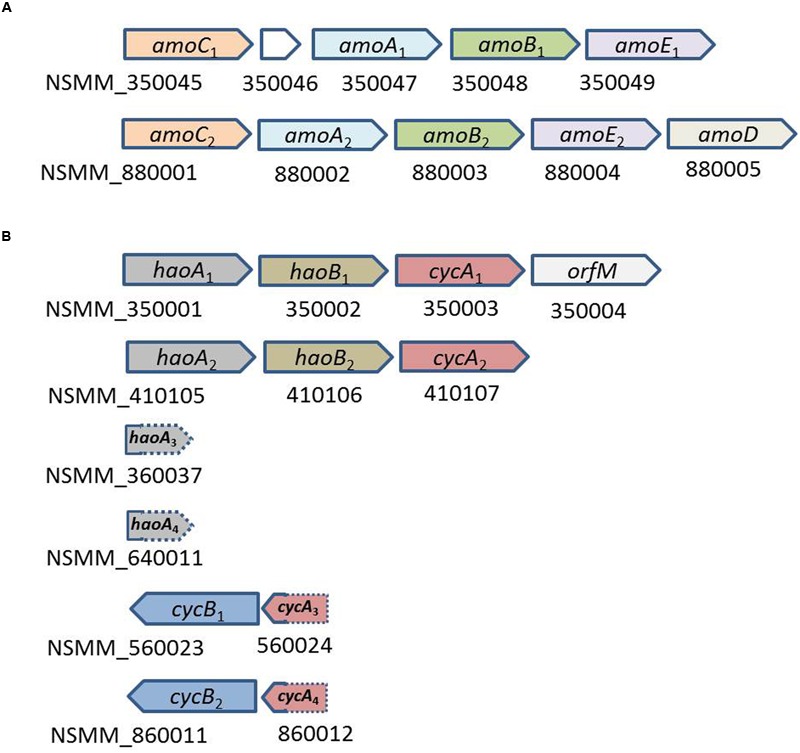
**Organization of the cluster of genes involved in the ammonia oxidation of *N. mobilis* Ms1. (A)** Cluster of genes encoding ammonia monooxygenase; NSMM_350046, the length of 159 bp, is the gene encoding protein with unknown function **(B)** cluster of genes encoding hydroxylamine oxidoreductase and associated cytochromes. The separate fragments *haoA*_3_, *haoA*_4_, *cycA*_3_, and *cycA*_4_, with the sequence length of 117, 165, 108, and 129 bp, respectively, were shorter than their respective version in the operon, and these were located at the end of the contigs and 3′ ends of the *haoA*_3_ and *haoA*_4_, and 5′ ends of the *cycA*_3_ and *cycA*_4_ were not included in the sequence of the contigs.

*N. mobilis* Ms1 uses ammonia as an energy source in the same manner as other AOB. The genome of *N. mobilis* Ms1 contains two copies of the gene cluster encoding ammonia monooxygenase (*amoCAB*), followed by two copies of the *amoED* operon in which *amoD* in the first operon was not identified (**Figure 6A**; **Table 2**). *N. mobilis* Ms1 contains the operons encoding hydroxylamine oxidoreductase with associated cytochromes c554 and c_M_552 (*haoAB-cycAB*) in two copies, neither of which includes *cycB*, whereas other AOB genomes contain two to three copies of the complete *haoAB-cycAB* operons (**Figure 6B**; **Table 2**) (Chain et al., 2003; Stein et al., 2007; Suwa et al., 2011). Unlike other AOB, the two small *haoA* fragments, which are distantly located from the *haoAB-cycA* operons and two copies of *cycB*-fragment *cycA* operon, but not adjacent to *hao*, were also identified in the *N. mobilis* Ms1 genome. The additional *amoC* singleton gene, which has been found in most other AOB genomes, was not identified in *N. mobilis* Ms1. As in other AOB, except *Nitrosomonas* sp. Is79 (Bollmann et al., 2013), the *N. mobilis* Ms1 genome contains an AOB-specific red-copper protein, nitrosocyanin, which has been proposed to be involved in electron transport, although the detailed vital function of this protein has not been identified (Klotz and Stein, 2011; Stein et al., 2013).

Ammonia-oxidizing bacteria are known to produce nitric oxide (NO) and nitrous oxide (N_2_O), a greenhouse gas, from the hydroxylamine oxidation (a part of ammonia oxidation), nitrifier denitrification (dissimilatory nitrite reduction) and NO detoxification (nitrosative stress) pathways (Klotz and Stein, 2011). The genes encoding copper-containing nitrite reductase (*NirK*), membrane-bound cytochrome c nitric oxide reductase cNOR (*norCBQD*), heme-copper nitric oxide reductase sNOR (*norSY-SenC*), cytochrome c′ beta (*cytS*) and cytochrome P460 (*cytL*), all of which are supposed to be involved in the oxidation of hydroxylamine, the nitrifier partial denitrifying process and NO-detoxification, were identified in the *N. mobilis* Ms1 genome, whereas one or both types of nitric oxide reductase were not identified in the *Nitrosomonas* sp. AL212 and *Nitrosomonas* sp. Is79 genomes (**Table 2**) (Klotz and Stein, 2011; Suwa et al., 2011; Bollmann et al., 2013). Unlike other AOB, the gene encoding nitric oxide reductase activation protein (*norD*) did not cluster with *norCBQ* in the *N. mobilis* Ms1 genome (**Table 2**). Overall, the gene contents and the organization of the genes involved in *N. mobilis* Ms1 were distinct compared to other AOB genomes.

## Conclusion

The key physiological and genomic properties of *N. mobilis* Ms1 were investigated in this study. The temperature and pH ranges and the growth rate of *N. mobilis* Ms1 were within the ranges of other *Nitrosomonas* species in the *Nitrosomonas europaea*/*Nitrosomonas mobilis* lineage, supporting the previous findings regarding the codominant habitat of *N. mobilis*-like bacteria with *N. europaea–N. eutropha*-like bacteria in wastewater treatment systems. In addition, *N. mobilis* Ms1 can withstand the high ammonia and nitrite concentrations. These physiological findings reveal the habitat and ecophysiology of *N. mobilis* in the natural ecosystem. Because *N. mobilis* Ms1 can oxidize at high ammonium and nitrite concentrations, *N. mobilis*-like strains would be dominant in environments such as wastewater treatment plants where the ammonia and nitrite concentrations are rather high. The genomic information for *N. mobilis* Ms1 is the first genome sequence of *Nitrosomonas mobilis*-like bacteria. The genome analysis of *N. mobilis* Ms1 also indicated that this strain has distinct genome features compared with the other *Nitrosomonas* species, although other functional genes and unique gene characteristics of Ms1 still needed to be investigated. Overall, the results of the present study provide a better understanding of the ecophysiology of *Nitrosomonas mobilis* and other nitrifying bacteria, especially among the widely abundant *Nitrosomonas* species in both natural and wastewater treatment systems.

## Author Contributions

SMT, NU, HF, and ST designed the experiments. SMT performed all experiments. SMT, NU, HF, and ST analyzed the data. YS performed and wrote genome sequencing and assembly. SMT performed genome analysis. NU performed ANI analysis. SMT drafted the manuscript. SMT, NU, HF, YS, and ST read and edited the manuscript.

## Conflict of Interest Statement

The authors declare that the research was conducted in the absence of any commercial or financial relationships that could be construed as a potential conflict of interest.

## Footnotes

1 http://www.ezbiocloud.net/ezgenome/ani
